# Fulvestrant: an oestrogen receptor antagonist with a novel mechanism of action

**DOI:** 10.1038/sj.bjc.6601629

**Published:** 2004-03-05

**Authors:** C K Osborne, A Wakeling, R I Nicholson

**Affiliations:** 1 Departments of Medicine and Molecular & Cellular Biology, Baylor College of Medicine, Houston, TX, USA; 2 AstraZeneca Pharmaceuticals, Macclesfield, Cheshire, UK; 3 Tenovus Centre for Cancer Research, Welsh School of Pharmacy, Cardiff, UK

**Keywords:** fulvestrant, ‘Faslodex’, oestrogen receptor antagonist, mechanism of action, advanced breast cancer

## Abstract

Due to their favourable tolerability profiles, endocrine therapies have long been considered the treatment of choice for hormone-sensitive metastatic breast cancer. However, the oestrogen agonist effects of the available selective oestrogen receptor modulators, such as tamoxifen, and the development of cross-resistance between endocrine therapies with similar modes of action have led to the need for new treatments that act through different mechanisms. Fulvestrant (‘Faslodex’) is the first of a new type of endocrine treatment – an oestrogen receptor (ER) antagonist that downregulates the ER and has no agonist effects. This article provides an overview of the current understanding of ER signalling and illustrates the unique mode of action of fulvestrant. Preclinical and clinical study data are presented in support of the novel mechanism of action of this new type of ER antagonist.

New hormonal therapies with novel mechanisms of action that are not cross-resistant with the existing treatments make important additions to the repertoire of treatments for breast cancer. This enables additional endocrine agents to be used sequentially, with the aim of extending the effective duration of well-tolerated treatment before cytotoxic chemotherapy becomes necessary (Carlson, 2002).

Fulvestrant (‘Faslodex’) is the first of a new type of endocrine treatment – an oestrogen receptor (ER) antagonist that downregulates the ER and has no agonist effects. An understanding of ER signalling is essential to distinguish between the mode of action of fulvestrant and that of tamoxifen and the other selective ER modulators (SERMs). This article summarises the current knowledge of oestrogen signalling, and outlines the mechanism of action of fulvestrant.

## OESTROGEN SIGNALLING AS A TARGET FOR BREAST CANCER THERAPY

17*β*-oestradiol, the dominant circulating oestrogen, controls the growth of many breast tumours. Oestradiol is secreted by the ovaries in premenopausal women, but is also present at significant levels in postmenopausal breast tumours. In postmenopausal women, oestrogens are produced by aromatase-mediated conversion of androgens (originating from the adrenal glands and the ovaries) to oestrogens, in normal tissues (adipose tissue, muscle, liver, or brain) as well as in breast tumours (Buzdar, 2001).

The ER is expressed in the majority of breast tumours (Jonat and Maass, 1978; Lee and Markland, 1978; Paszko *et al*, 1978) and in a number of endocrine tissues including the normal breast, uterus and vagina, as well as in the pituitary and hypothalamus.

**Figure 1:**
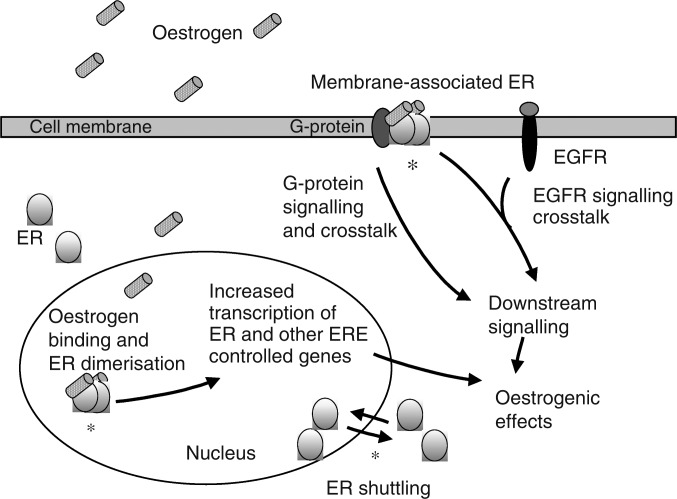
Cellular distribution and activity of the ER. The known mechanisms of fulvestrant intervention in ER signalling are indicated with an asterisk. EGFR=epidermal growth factor receptor; ERE=oestrogen response element; ER=oestrogen receptor.

Oestradiol binds to the ER with a high affinity and specificity and, once bound, the oestradiol/ER complex can exert its effects at both nuclear and cell membranous sites (Figure 1

). In the classical nuclear ER pathway of transcriptional control, the binding of oestradiol to the ER initiates dissociation of heat shock proteins from the ER, followed by receptor dimerisation and preferential nuclear localisation (Beato, 1989; MacGregor and Jordan, 1998).

The oestrogen–ER dimer complex binds to specific DNA sequences, the oestrogen response elements (EREs), which are situated in the regulatory regions of oestrogen-sensitive genes. Transcriptional control is mediated via two regions of the ER-designated activation functions AF1 and AF2, which recruit other proteins such as transcriptional co-activators and co-repressors to the transcriptional complex (Beato, 1989; Tsai and O'Malley, 1994; Horwitz *et al*, 1996; White and Parker, 1998). AF1 activity is regulated by growth factors that act via the mitogen-activated protein kinase (MAPK) pathway (Kato *et al*, 1995), while the AF2 domain is activated by oestrogen (Kumar *et al*, 1987). Both domains are required to be active for full oestrogen agonist activity. The ER mediates transcriptional regulation of a range of genes, directly or indirectly associated with proliferation, invasion, survival or angiogenesis in breast cancer.

To date, two ERs have been identified: the ‘classic’ ER*α* and the relatively more recently described ER*β* (Kuiper *et al*, 1996). These two ER subtypes have different tissue distributions (Speirs *et al*, 2002), different affinities and responsiveness to various SERMs (Ogawa *et al*, 1998), and are under different regulatory control (Katzenellenbogen and Katzenellenbogen, 2000). Oestrogen receptor*α* rather than ER*β* appears to be the predominant regulator of oestrogen-induced genes in breast cancer (Palmieri *et al*, 2002; Fuqua *et al*, 2003).

In addition to the classical ER signalling pathway, the ER can also undergo ‘crosstalk’ with growth factor and G-protein-coupled signalling pathways (Philips *et al*, 1993; Losel and Wehling, 2003) (Figure 1). For example, oestrogen can activate membrane-bound ER and, via G-protein activation, can then activate growth factor receptors such as the epidermal growth factor (EGF) receptor and human epidermal growth factor receptor 2 (HER2/neu) (Filardo, 2002; Johnston *et al*, 2003). In turn, the ER itself may be activated in a ligand-independent manner by other signalling molecules such as growth factors and protein kinases that control the phosphorylation state of the ER complex and play a part in regulating activity of the ER (Katzenellenbogen *et al*, 2000).

## THE NEED FOR ALTERNATIVE ENDOCRINE THERAPIES

In patients with hormone-sensitive advanced breast cancer, endocrine therapy is better tolerated than cytotoxic chemotherapy, while being equally effective (Buzdar, 2001). However, there are specific risks associated with endocrine treatments. For example, tamoxifen treatment is associated with a 2–4-fold increased risk of endometrial cancer (Early Breast Cancer Trialists' Collaborative Group, 1998), attributable to its oestrogen-like, partial agonist activity. The ‘Arimidex’, Tamoxifen Alone or in Combination (ATAC) trial showed a significantly greater incidence of ischaemic cerebrovascular events (2.1 *vs* 1.0%; *P*=0.0006) and venous thromboembolic events (3.5 *vs* 2.1%; *P*=0.0006) with tamoxifen, compared with the aromatase inhibitor (AI) anastrozole (ATAC Trialists' Group, 2002). A number of other antioestrogens grouped together under the term SERMS have also been associated with partial agonist properties (Johnston, 2001; Arun *et al*, 2002).

The AIs letrozole and exemestane may have an unfavourable effect on plasma lipid levels, and androgenic side effects have been reported with exemestane (Buzdar, 2003). Megestrol acetate, historically the most widely used progestin, is associated with weight gain and fluid retention (Espie, 1994) and the high-dose oestrogen diethylstilboestrol is commonly associated with nausea, oedema, vaginal bleeding and cardiac problems (Peethambaram *et al*, 1999).

The sequential use of well-tolerated hormonal therapies has become common clinical practice for the treatment of advanced breast cancer, where maintenance of quality of life is a primary aim. For this to be effective, it is necessary that the mechanism of action of newer agents differ from those previously used. This prerequisite prevents the sequential use of therapies belonging to the same class, and that therefore demonstrates cross-resistance with each other. Therefore, for some time, a search has been under way for an antioestrogen that lacks partial agonist properties and that has a mechanism of action different from tamoxifen (Wakeling and Bowler, 1988).

## FULVESTRANT: A POTENT ER ANTAGONIST WITH A NOVEL MECHANISM OF ACTION

### Blockade of oestrogen action via ER antagonism

**Figure 2:**
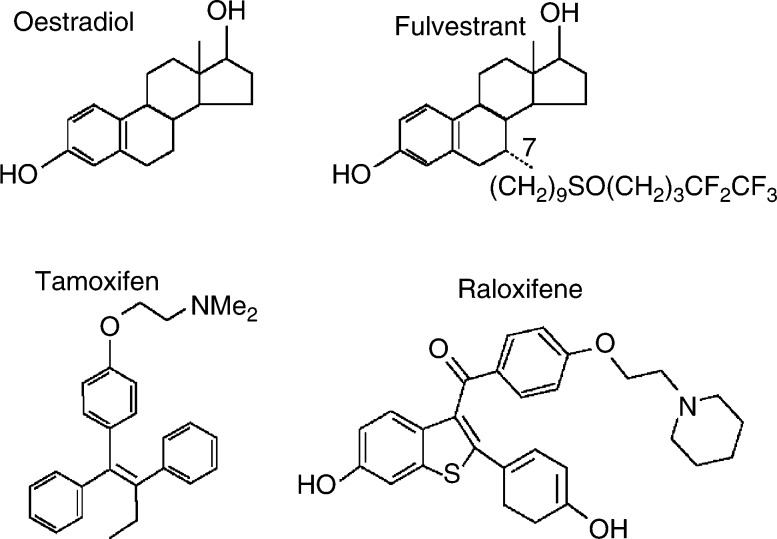
Structure of 17*β*-oestradiol, fulvestrant, tamoxifen and raloxifene.

Fulvestrant is a 7*α*-alkylsulphinyl analogue of 17*β*-oestradiol, which is distinctly different in chemical structure from the nonsteroidal structures of tamoxifen, raloxifene and other SERMs (Figure 2

). Fulvestrant competitively inhibits binding of oestradiol to the ER, with a binding affinity that is 89% that of oestradiol (Wakeling and Bowler, 1987). This is markedly greater than the affinity of tamoxifen for the ER (which is 2.5% that of oestradiol) (Wakeling and Bowler, 1987; Wakeling *et al*, 1991).

**Figure 3:**
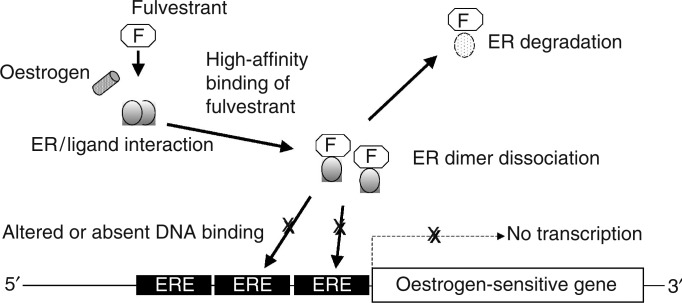
Mechanism of action of fulvestrant at the level of transcriptional regulation. ERE=oestrogen response element; ER=oestrogen receptor; F=fulvestrant.

Fulvestrant–ER binding impairs receptor dimerisation, and energy-dependent nucleo-cytoplasmic shuttling, thereby blocking nuclear localisation of the receptor (Fawell *et al*, 1990; Dauvois *et al*, 1993) (Figure 3

). Additionally, any fulvestrant–ER complex that enters the nucleus is transcriptionally inactive because both AF1 and AF2 are disabled. Finally, the fulvestrant–ER complex is unstable, resulting in accelerated degradation of the ER protein, compared with oestradiol- or tamoxifen-bound ER (Nicholson *et al*, 1995b). This downregulation of cellular ER protein occurs without a reduction in ER mRNA. Thus, fulvestrant binds, blocks and accelerates degradation of ER protein, leading to complete inhibition of oestrogen signalling through the ER (Osborne *et al*, 1995; Wakeling, 1995,^2000^; Wardley, 2002).

### Fulvestrant has no demonstrable agonist activity

The disruption of both AF1 and AF2 sites means that, in contrast to the SERMs such as tamoxifen which fail to inhibit AF1 activity and thereby have partial oestrogen agonist activity, fulvestrant has no oestrogen agonist activity in animals or man. This lack of agonist activity has been demonstrated in numerous animal models of oestrogen action. Thus, in immature female rats, fulvestrant, unlike tamoxifen, was completely devoid of uterotrophic activity. Correspondingly, co-administration of fulvestrant with either oestradiol or tamoxifen blocked the maximal and partial uterotrophic activity of oestradiol or tamoxifen, respectively, in a dose-dependent and complete manner (Wakeling *et al*, 1991). In contrast, co-administration of tamoxifen and oestradiol only partially blocks the uterotrophic action of oestradiol. In primate studies, fulvestrant inhibited oestradiol-induced increases in the volume of the endometrium; the rate and extent of endometrial involution in fulvestrant-treated monkeys was similar to that seen following oestrogen withdrawal (Dukes *et al*, 1992). In a Phase I trial involving 30 postmenopausal volunteers, fulvestrant 250 mg (intramuscular (i.m.) injection) demonstrated no agonist effects on the human endometrium during the 14-day period of administration. In addition, the antagonistic effects of fulvestrant were confirmed by a significant inhibition of the oestrogen-stimulated thickening of the endometrium compared with placebo (*P*=0.0001) (Addo *et al*, 2002).

## BIOLOGICAL EFFECTS AND LACK OF CROSS-RESISTANCE WITH TAMOXIFEN

### Preclinical antitumour activity and effects on ER signalling

Studies in the MCF-7 human breast cancer cell line have shown that fulvestrant significantly suppresses cellular levels of ER protein (McClelland *et al*, 1996a) and inhibits ER-induced expression of the progesterone receptor (PgR), the oestrogen-regulated protein pS2 and cathepsin D more strongly than tamoxifen (Nicholson *et al*, 1995a). In a study of global gene expression in MCF-7 cells, after supplemental oestrogen, a subset of ER-responsive genes upregulated by oestrogen were selected, and the effects of fulvestrant and tamoxifen were analysed by microarray expression profiling and Northern blot analysis (Inoue *et al*, 2002). For most of these genes, oestrogen-regulated expression was completely abolished by fulvestrant. In contrast, in the presence of tamoxifen, some genes remained, in part, transcriptionally responsive to oestrogen (Inoue *et al*, 2002). Similarly, in MCF-7 tumour xenografts, fulvestrant has also been shown to be more effective than tamoxifen in reducing cellular levels of the ER and PgR; expression levels of other oestrogen-regulated genes pLIV1 and pS2 were also greatly reduced (Osborne *et al*, 1994,Osborne *et al*, 1994, 1995).

Fulvestrant also blocks ER-mediated effects in the MCF-7 cell line by decreasing the levels of transforming growth factor *α* (TGF*α*), thereby reducing ‘crosstalk’ between these pathways (Nicholson *et al*, 1995a). Furthermore, in rat adipocytes, physiological concentrations (0.1–10 nM) of oestrogen have been shown to rapidly activate the p42/p44 MAPK independently of transcriptional activation. This effect is also blocked by fulvestrant (Dos Santos *et al*, 2002).

Fulvestrant is a more effective growth inhibitor of ER-positive MCF-7 human breast cancer cells than tamoxifen, producing an 80% reduction in cell numbers under conditions where tamoxifen achieved a maximum of 50% inhibition (Wakeling and Bowler, 1987). Flow cytometry of MCF-7 cells showed fulvestrant to be more effective than tamoxifen in increasing the proportion of cells in G_0_/G_1_ and decreasing the proportion of cells capable of continued DNA synthesis (Wakeling and Bowler, 1987; Wakeling *et al*, 1991). Importantly, fulvestrant has also demonstrated antitumour activity in tamoxifen-resistant MCF-7/TAM^R−1^ cell lines, confirming a lack of cross-resistance between tamoxifen and fulvestrant (Hu *et al*, 1993; Lykkesfeldt *et al*, 1994). At fulvestrant concentrations of 5–10 nmol l^−1^, cell growth of tamoxifen-resistant MCF-7 cells was completely inhibited. Compared with tamoxifen, fulvestrant was 150 times more effective at inhibiting cell growth in the tamoxifen-sensitive parental line, and 1540 times more effective in the tamoxifen-resistant variant cell line (Hu *et al*, 1993). Furthermore, in later preclinical studies, fulvestrant-resistant MCF-7 cells demonstrated no resistance to tamoxifen, with sensitivity similar to that of the parental cell line (Lykkesfeldt *et al*, 1995).

*In vivo*, the antitumour activity of fulvestrant was first demonstrated in two models of human breast cancer in nude mice. In one of these models, the growth of MCF-7 tumour xenografts, supported by continuous treatment with oestradiol, was completely blocked for at least 4 weeks following a single injection of fulvestrant 5 mg (Osborne *et al*, 1995). Similar reductions in growth were seen in the Br10 human tumour model (Wakeling *et al*, 1991). In other studies in nude mice bearing MCF-7 xenografts, fulvestrant suppressed the growth of established tumours for twice as long and tumour growth was delayed to a greater extent than was observed with tamoxifen treatment. Tamoxifen-resistant breast tumours, which grew in nude mice after long-term treatment with tamoxifen, remained sensitive to growth inhibition by fulvestrant (Osborne *et al*, 1994).

### Antitumour activity and effects on ER signalling in patients with breast cancer

The biological and antitumour effects of fulvestrant have also been evaluated in several trials involving postmenopausal women with primary breast cancer. The effects of daily i.m. injections of short-acting fulvestrant (either 6 or 18 mg) for 7 days prior to surgery for primary breast cancer were compared with no pretreatment controls in 56 postmenopausal women (DeFriend *et al*, 1994). In patients with ER-positive (ER+) tumours (28/56), fulvestrant caused a significant reduction in median ER index (0.73 *vs* 0.02 pre- and post-treatment, respectively; *P*<0.001) and almost abolished PgR expression; the median PgR index was reduced from 0.50 to 0.01 post-treatment (*P*<0.05; *n*=37) in ER+ tumours. This reduction in cellular ER protein occurred without a concurrent reduction in ER mRNA levels (McClelland *et al*, 1996b). Fulvestrant caused a significant reduction in pS2 expression and tumour proliferation. pS2 expression was reduced from 7 to 1% after treatment (*P*<0.05; *n*=37) and the proliferation marker Ki67 was reduced from 3.2 to 1.1% following fulvestrant treatment (*P*<0.05) (DeFriend *et al*, 1994).

**Figure 4:**
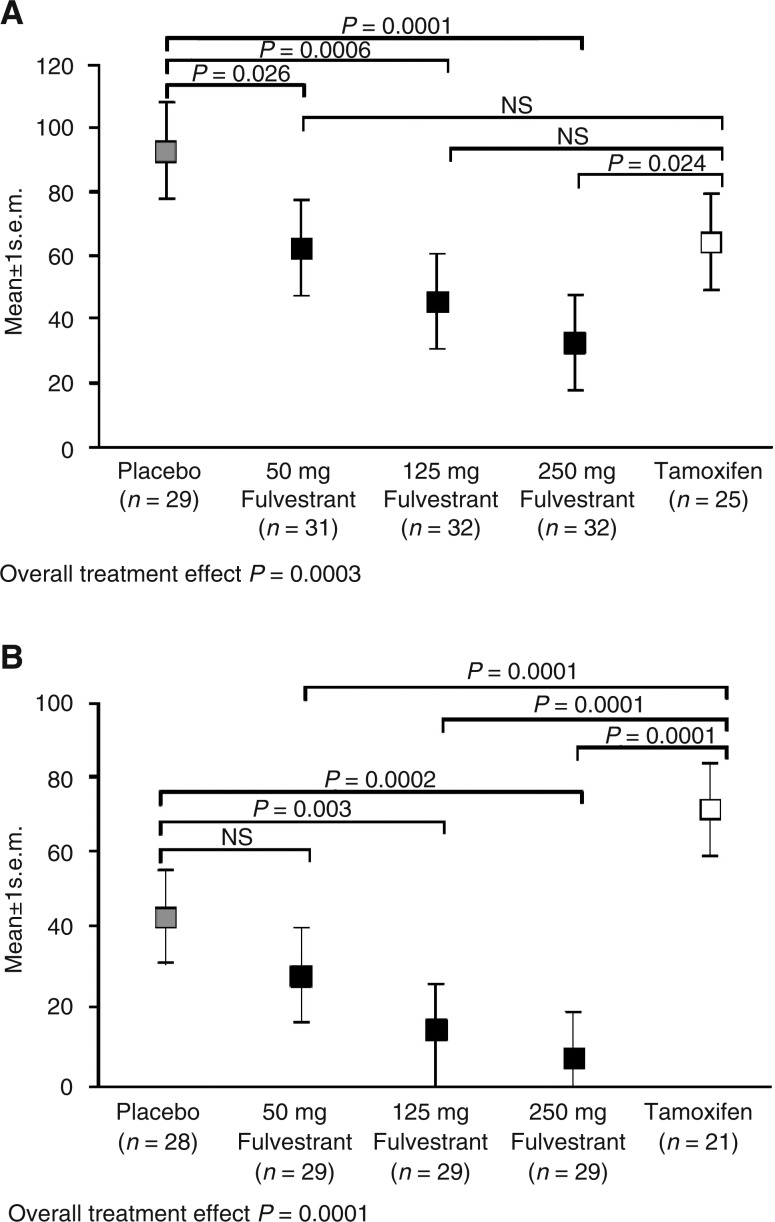
Mean (**A**) ER and (**B**) PgR levels after a single i.m. injection of 50, 125, or 250 mg fulvestrant, 20 mg tamoxifen, or placebo. Reproduced with the permission of Cancer Research (Robertson *et al*, 2001).

In a subsequent study that compared the effects of a single dose of long-acting fulvestrant (50, 125, or 250 mg), continuous daily tamoxifen, or placebo for 14–21 days in patients with primary breast tumours, all fulvestrant doses produced statistically significant reductions in ER expression compared with placebo (50 mg: 32% reduction, *P*=0.026; 125 mg: 55% reduction, *P*=0.0006; 250 mg: 72% reduction, *P*=0.0001). At the higher 250 mg dose, the fulvestrant-induced reduction was significantly greater than that observed with tamoxifen (*P*=0.024) (Robertson *et al*, 2001). Significant reductions in PgR expression were also observed at the fulvestrant 125 mg (*P*=0.003) and 250 mg (*P*=0.0002) doses compared with placebo. In contrast, tamoxifen resulted in a significant increase in PgR expression relative to placebo, a finding attributed to its partial agonist effects and further emphasising the differences in mode of action between fulvestrant and tamoxifen (Robertson *et al*, 2001) (Figure 4

).

Fulvestrant produced significant dose-dependent reductions in Ki67 compared with placebo (50 mg: *P*=0.046; 125 mg: *P*=0.001; 250 mg: *P*=0.0002), although there were no differences in Ki67 between fulvestrant and tamoxifen (Robertson *et al*, 2001). The cell turnover index (CTI) is a composite measurement of both cell proliferation and apoptosis, and provides a useful indicator of drug action on breast tumour growth. In the same study, patients receiving fulvestrant 250 mg showed a significant reduction in the CTI compared with those who received placebo (*P*=0.0003) and tamoxifen (*P*=0.026). The effect on CTI with tamoxifen was not significantly different from that with placebo (Bundred *et al*, 2002).

Taken together with the preclinical data, these findings emphasise the differences in mode of action and the lack of cross-resistance between the SERMs and fulvestrant, which has latterly been supported by phase III data, demonstrating the efficacy of fulvestrant in patients with tamoxifen-resistant disease.

## CONCLUSIONS

Fulvestrant is a new type of endocrine treatment – an ER antagonist with a novel mode of action. Fulvestrant disrupts ER dimerisation and nuclear localisation, completely blocking ER-mediated transcriptional activity and accelerating receptor degradation. Consequently, fulvestrant also blocks the activity of oestrogen-regulated genes associated with breast tumour progression, invasion, metastasis and angiogenesis. The antitumour effects of fulvestrant have been demonstrated both in preclinical studies and in clinical trials, using a number of prognostic and predictive markers. This new type of endocrine therapy has no oestrogen agonist effects, and lacks cross-resistance with other antioestrogens. Antioestrogens with novel mechanisms of action such as fulvestrant represent a valuable second-line treatment option for postmenopausal women with hormone-sensitive advanced breast cancer, who have progressed on prior tamoxifen therapy. Fulvestrant and other new endocrine therapies may also provide opportunities for a longer treatment period with well-tolerated endocrine therapy before the need for cytotoxic chemotherapy.
